# Effects of Resveratrol on the Mechanisms of Antioxidants and Estrogen in Alzheimer's Disease

**DOI:** 10.1155/2019/8983752

**Published:** 2019-03-20

**Authors:** Danli Kong, Yan Yan, Xiao-Yi He, Huihuang Yang, BiYu Liang, Jin Wang, Yuqing He, Yuanlin Ding, Haibing Yu

**Affiliations:** ^1^Department of Epidemiology and Health Statistics, Public Health School of Guangdong Medical University, Dongguan 523808, Guangdong, China; ^2^Academic College of Guangdong Medical University, Dongguan 523808, Guangdong, China; ^3^Systems Biology Research Institute of Guangdong Medical University, Dongguan 523808, Guangdong, China; ^4^Department of Science and Technology, Guangdong Medical University, Dongguan 523808, Guangdong, China

## Abstract

**Objective:**

To observe the effects of resveratrol (Res) on the antioxidative function and estrogen level in an Alzheimer's disease (AD) mouse model.

**Methods:**

First, we examined the effects of Res on an AD mice model. SAMP8 mice were selected as the model, and normal-aging SAMR1 mice were used as the control group. The model mice were randomly divided into three groups: a model group, high-dose Res group (40mg/kg, intraperitoneal (ip)), and low-dose Res group (20mg/kg, ip). After receiving medication for 15 days, the mice were subjected to the water maze test to assess their spatial discrimination. The spectrophotometric method was used to detect the activity of superoxide dismutase (SOD), glutathione peroxidase (GSH-Px), and catalase (CAT) as well as the malondialdehyde (MDA) content. Quantitative PCR (q-PCR) was used to detect SOD, GSH-Px, CAT, and heme oxygenase-1 (HO-1) mRNA level changes. Western blot analysis detected HO-1 and Nrf2 protein expression. Second, we researched the effect of Res on the estrogen level in the SAMP8 model mice. The model mice were randomly divided into four groups: a model group, estrogen replacement group (0.28 mg/kg, intramuscular (im), estradiol benzoate), high-dose Res group (5 mg/kg, im), and low-dose Res group (2.5 mg/kg, im). The mice were injected, once every three days, for 5 weeks. Q-PCR was used to detect brain tissue mRNA expression changes. Western blot analysis detected ER*α*, ER*β*, and ChAT protein expression. An enzyme-linked immunosorbent assay (ELISA) kit was used to detect the expression of E2 and amyloid *β* protein (A*β*) in brain tissue.

**Results:**

Compared with the control treatment, Res could improve the spatial abilities of the mice to a certain extent and also increase the expression of SOD, GSH-Px, CAT, and HO-1 at the mRNA level (P<0.05). In addition, enhanced SOD, GSH-Px, and CAT activities and HO-1 protein levels and decreased MDA content (P<0.05) were detected in the brain tissue of the Res-treated mice. The cytoplasmic Nrf2 content in the Res-treated mice was also decreased while the nuclear Nrf2 content and the nuclear translation rate of Nrf2 were increased (P<0.05). Res could decrease the expression of ER*β* in the brain tissue at the mRNA and protein levels and the expression of A*β* in the brain tissue at the protein level. Res could also increase the mRNA and protein expression of ER*α* and ChAT and the protein expression of estradiol in the brain tissue.

**Conclusion:**

Res can increase the antioxidant capacity of AD models through the Nrf2/HO-1 signaling pathway. In addition, Res can enhance estrogen levels in an AD model. These findings provide a new idea for the treatment of AD.

## 1. Introduction

Alzheimer's disease (AD) is a degenerative disease of the nervous system that occurs in the brain and causes memory decline, decreased intelligence, vague language, and strange behavior. It is the main cause of dementia in adults [1]. Its characteristic pathological changes are a reduction in the number of neurons and deposition of senile plaques or neurofibrillary tangles [2] in the cerebral cortex and hippocampus. Among all AD patients, approximately 60% have sporadic AD, and the exact pathogenesis of AD is still incompletely understood.

Resveratrol (Res), also named resveratrol, has a structure similar to that of estrogen diethylstilbestrol. Res is mainly derived from plants such as peanuts, grapes (red wine), Polygonum cuspidatum, and mulberries. A study found that Res is a naturally occurring biological polyphenol and a natural antioxidant [3]. Oxidative stress refers to a pathological intracorporeal state in which the amount of free radicals or other products exceeds the body's antioxidant capacity. When there is an imbalance in redox reactions in vivo, excess reactive oxygen species (ROS) and reactive nitrogen species (RNS) are generated. In addition, in the context of an excess of metal ions, the Fenton reaction forms hydroxyl radicals, which can potentially cause damage to the body [4]. Numerous lines of evidence indicate that the loss of oxidative stress or antioxidant defense enzymes caused by an increase in ROS and RNS levels may also play an important role in AD. For the past few years, the theory of amyloid *β*- protein (A*β*) has been widely recognized. There are also more reports on A*β*, which state that A*β* participates in the pathogenesis of AD through oxidative stress and that the effect of therapeutic antioxidant drugs on AD is mediated by acting on A*β*. It has been suggested that an intervention targeting A*β*-related oxidative stress may be an important strategy in the prevention and treatment of AD [5].

The occurrence and development of AD are associated with low estrogen levels. A study found that the levels of estrogen in postmenopausal women in vivo are extremely low. In addition, female AD patients exhibit more severe cognitive impairment than male patients, suggesting that endogenous and exogenous estrogen can affect the development of AD through a variety of mechanisms [6]. Experimental evidence also indicates that estrogen can significantly increase neuronal activity and reduce A*β* aggregation, which suggests that estrogen can be used to prevent or even treat AD [7]. This study aimed to investigate whether Res can ameliorate the progression of AD by improving oxidative stress and estrogen levels in an AD model and is expected to provide a basis for the development of new drugs and clinical treatments for AD.

## 2. Materials

### 2.1. Animals

A total of 70 fast-aging dementia model SAMP8 mice and 20 normal-aging SAMR1 mice were used in this study. All the mice were male, eleven months old, and approximately 21g in weight. The mice were provided by Guangdong Medical Experimental Animal Center, certification number: SCXK(Guangdong)2013-0002. The mice were housed with free feeding and drinking water and a normal light cycle.

### 2.2. Drugs and Reagents

The following reagents were purchased: Res (Sigma-Aldrich, purity ≥99%, Item No. 501360); estradiol benzoate (Dalian Meilun Biotechnology Co., Ltd., purity ≥99%, Item No. MB1099); Trizol Reagent (Invitrogen, USA, Item No. 15596018); ReverTra Ace qPCR RT Kit, and SYBR Green qPCR Master Mix (TOYOBO, Japan, Item No. FSQ101 and SCQ101, respectively). Superoxide dismutase (SOD) (Item No. A001-2), glutathione peroxidase (GSH-Px) (Item No. A005), catalase (CAT) (Item No. A007-1), and malondialdehyde (MDA) (Item No. A003-1) kits were purchased from Nanjing Jiancheng Bioengineering Institute. A mouse estradiol enzyme-linked immunosorbent assay (ELISA) kit (Shanghai Kai Bo Biochemical Reagents Co., Ltd., Item No. BH3788), mouse A*β* ELISA kit (Shanghai Ke Min Biotechnology Co., Ltd., Item No. BH8906), anti-ER*α* primary antibody (Santa Curz Biotechnology, Item No. SC-542), anti-ER*β* antibody (Santa Cruz Biotechnology, Item No. SC-390243), and anti-ChAT antibody (Abcam, Item No. ab18736) were also purchased. SOD, CAT, and MDA primers were all designed and synthesized by TaKaRa. An anti-HO-1 primary antibody (Wuhan Boster Biological Engineering Co., Ltd., Item No. BA0605) and an anti-*β*-actin antibody (Abcam, Item No. ab8227) were also obtained.

### 2.3. Instruments

Instruments are a mouse water maze instrument (Chinese Academy of Medical Sciences), Tanon-1600 Gel image analysis system (Shanghai Tianneng Technology Co., Ltd.); 5810R Desktop High-speed refrigerated centrifuge (Eppendorf, Germany); Synergy HT Multifunctional microplate reader (Bio-Tek, USA); BIO-RAD electrophoresis and transfer systems (BIO-RAD, USA); and 7500 Fluorescence quantitative PCR instrument (ABI, USA).

## 3. Methods

### 3.1. Grouping and Dosing the Antioxidant Experimental Animals

(1) Before the experiment, animals were adaptively domesticated for 1 week and allowed food ad libitum. The experiment is comprised of a SAMR1 control group containing normal-aging mice, a SAMP8 model group, a high-dose Res group, and a low-dose Res group, with 10 mice/group. The experimental groups were given Res (40 mg/kg or 20 mg/kg) by gavage at 9:00 am every day for 15 consecutive days, simultaneously. The model group and the control group were given olive oil at a dose determined by the mouse body weight.

(2)* The Morris water maze* was used to measure for the spatial memory ability of the mice. The experiment began at 2 h after the last administration. The water maze had a universal cylindrical shape. The diameter of the water tank was 100 cm, the height was 40 cm, and the water depth was 25 cm. During the experiment, the temperature of the water was controlled at 22-24°C. Black ink was added to the water to make the water black and opaque. The edge of the pool wall was evenly distributed among four differently shaped markers, and then the pool was divided into four quadrants with these markers. The first quadrant had a middle height of 14 cm, a platform diameter of 6 cm, and a water depth of 2 cm. Each test required a variety of environmental factors outside the maze, such as lighting, laboratory positions, and reference objects, to be consistent and quiet. Before the experiment, the mice were placed on the platform to adapt for 10 s, and then the mice were randomly moved from the different quadrant walls into the pool. After the mice stopped on the platform for 5s, the test was terminated. If the mouse could not find the stage within 120 s, they were guided to the platform to adapt for 10 s and eventually returned to their cage. The mice were placed in the swimming pool 4 times a day at intervals of 1 hour, and navigation training required 4 days. Measuring platform escape latency evaluated the spatial memory ability of the mice.

(3) Determination of brain SOD, GSH-Px, CAT activities, and MDA content: after the end of the last administration, the mice were sacrificed by dislocating the cervical vertebra, and the brain tissue was removed. According to the manufacturer's instructions, the absorbance of each well was determined by a microplate reader, and the activity was calculated according to a formula. The SOD, GSH-Px, CAT activities, and MDA content were measured.

(4)* Real-time fluorescent quantitative* PCR (q-PCR) detected the mRNA expression of SOD, GSH-Px, CAT, and HO-1 in the brain tissue. The Trizol method was used to extract total RNA from the brain tissue. An ultraviolet (UV) spectrophotometer detected the purity of the RNA, and a 1.5% sepharose gel tested the RNA integrity. The RNA samples underwent reverse transcription to synthesize cDNA templates, which then underwent PCR amplification. The reaction conditions were predenaturation at 94°C for 3 min; denaturation at 94°C for 30 s; refolding at 68°C for 40 s; extension at 72°C for 2 min for 35 cycles; and extension at 72°C for 10 min. According to the standard curve amplification efficiency consistency, the relative expression of HO-1, SOD, GSH-Px, and CAT in samples was analyzed by the 2-ΔΔCt method.

(5) Western blot analysis detected the expression of HO-1, Nrf2, and Kelch-like ECH-associated protein 1 (Keap1). Animal brain tissue from each group was added to 500*μ*l of RIPA lysis buffer per 100 mg of tissue and centrifuged at 10,000r-·min-1 for 5min after full lysis. The liquid supernatant was collected, the total protein concentration was measured by the BCA method, and loading buffer was added at a 1:1 ratio and then boiled in boiling water for 5 min. After taking 50 *μ*g of total protein for 12% SDS-polyacrylamide gel electrophoresis, transferring the proteins to a 5% skim milk powder was then used to block the membrane at room temperature for 2 hours. The membrane was washed 3 times with TBST buffer solution for 5 min each time. Then, a primary antibody solution (anti-HO-1 antibody, 1:800) was added and incubated at 4°C overnight. The membrane was washed in the same way, and an HRP-labeled secondary antibody solution (1:15,000) was added and incubated at room temperature for 2 hours. After washing in the same way, an added ECL luminescence reagent was added, and the samples were placed in a cassette for processing, developing, and fixing.

### 3.2. Estrogen-Induced Experimental Animals Were Grouped and Treated

Experimental mice were randomly divided into five groups as follows: a control group (CON), model group (M), estrogen replacement group (M+E), high-dose Res group (M+Res H), and low-dose group (M+Res L), with 10 mice in each group. At the beginning of the experiment, the estrogen replacement group received an intramuscular injection of 0.28 mg/kg estradiol benzoate. The high-dose Res group was intramuscularly injected with 5 mg/kg Res, and the low-dose Res group was intramuscularly injected with 2.5 mg/kg Res every 3 days. In addition, the model group and control group were injected with olive oil at a dose determined by the mouse body weight for 5 weeks.

(1) Q-PCR was used to detect ER*α*, ER*β*, and ChAT expression changes. Total RNA was extracted from the brain tissue with the Trizol method. The purity of the extracted RNA was determined with a UV spectrophotometer, and the integrity of the extracted RNA was tested with a 1.5% sepharose GIO-gel. The RNA samples were subjected to a reverse transcription reaction to synthesize cDNA templates, which were then subjected to PCR amplification. The reaction conditions were as follows: predegeneration at 95°C for 3 min; denaturation at 95°C for 30 s; annealing at 60°C for 30 s; and elongation at 72°C for 2 min for 35 cycles in total. The samples were incubated at 72°C for 10 min. According to the consistency of the standard curve amplification efficiency, the relative expression levels of ER*α*, ER, and ChAT in the samples were analyzed using the 2-ΔΔ Ct method, and the primer sequences are shown in Table 1.

(2) Western blot analysis was used to assess changes in protein expression of ER*α*, ER*β*, and ChAT. Animal brain tissue from each group was added to 500 *μ*l of RIPA lysis buffer per 100 mg of tissue and centrifuged at 10,000 r·min^−1^ for 5 min after full lysis. After taking 50 *μ*g of total protein for 12% SDS-polyacrylamide gel electrophoresis, the proteins were transferred to a membrane, and 5% skim milk powder was used to block the membrane for 2 hours at room temperature. The membrane was washed with TBST buffer solution 3 times for 5 min each time, and a primary antibody solution (ER*α*, 1:200; ER*β*, 1:2000 or ChAT, 1:1000) was added and incubated at 4°C overnight. The membrane was washed in the same manner, and the corresponding HRP-labeled secondary antibody solution (1:15,000) was added and incubated at room temperature for 2 hours. After washing the membrane in the same way, an ECL luminescence reagent was added, and the membrane was placed into a cassette for processing, developing and fixing.

(3) A*β* and estradiol expression changes were measured by ELISA. After the last drug delivery, the mice in each group were sacrificed, and the brain tissue was removed. According to the kit instructions, the absorbance of each well was measured by ELISA, and a standard curve was generated from the standards to calculate the A*β* and estradiol levels.

## 4. Statistical Analysis Was Performed

All data are expressed as the mean±standard deviation (±s). One-way analysis of variance and least significant difference (LSD) tests were performed using SPSS13.0 for Windows statistical software, and differences were considered statistically significant at P<0.05.

## 5. Results 

### 5.1. Water Maze Experiment Results

Compared with the control group, the model group had significantly prolonged latency time and swimming distance. The M+Res H group, compared with the M group, had a significantly shortened the latency time and swimming distance. There was no statistical difference between the M+Res L and M groups (Table 2).

### 5.2. Real-Time Fluorescent q-PCR Detection of SOD, GSH-Px, CAT, and HO-1 mRNA Expression in Brain Tissue

**Figure 1:**
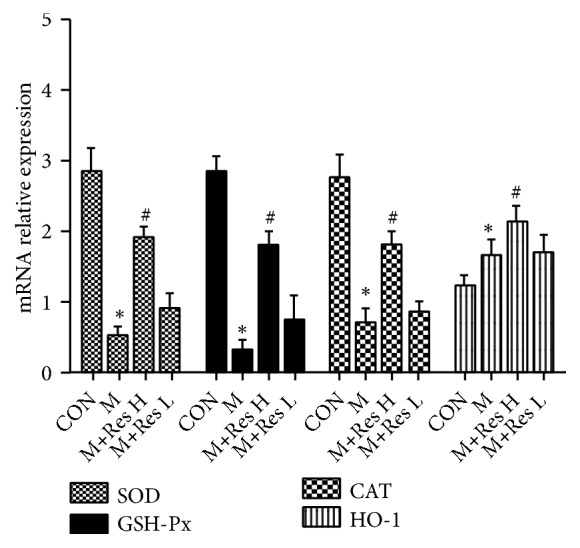
Changes in the brain tissue expression of antioxidant response molecules among the groups (±s, n=10). Note: compared with the CON group, *∗P* <0.05; compared with the model group, ^#^*P *<0.05.

As shown in Figure 1, the mRNA expression of SOD, GSH-Px, and CAT was decreased in the model group compared with the control, and HO-1 mRNA expression was increased in the model group. Compared with the model group, the high-dose Res group exhibited increased mRNA expression of SOD, GSH-Px, CAT, and HO-1.

### 5.3. Effect of Res on the Antioxidant Abilities of the AD Model

From Table 3, we can see that, compared with those of the control group, the SOD, GSH-Px, CAT, and other activities of the model group have decreased to varying degrees, and the MDA content of the model group increased significantly.

Compared with the model group, the high-dose Res group can increased SOD, GSH-Px, and CAT activities and reduced MDA content. However, compared with the model group, the low-dose Res group showed no statistical difference. This suggests that a high dose of Res can significantly improve the antioxidant capacity of the AD model (Table 3).

### 5.4. Western Blot Detection of HO-1 and Nrf2 Expression

**Figure 2:**
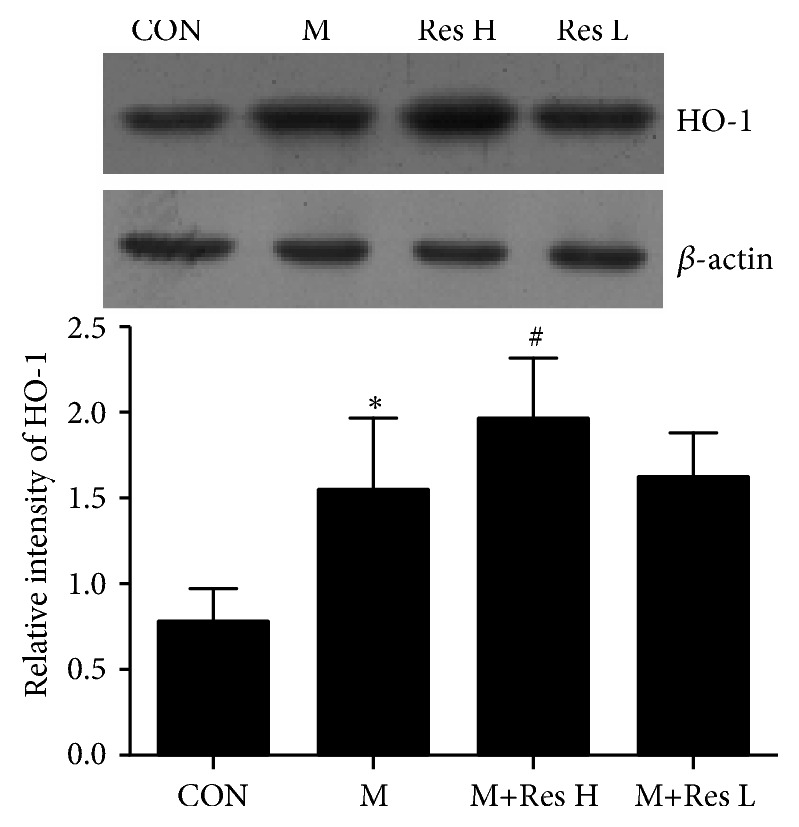
Changes in the HO-1 protein expression in the brain tissue from each group (±s, n=10). Note: compared with the CON group, *∗*P <0.05; compared with the model group, #P <0.05.

**Figure 3:**
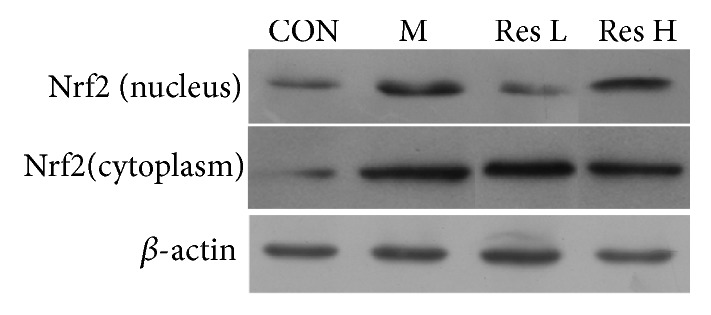
Effect of Res on Nrf2 protein expression in the AD model brain tissue.

As shown in Figures 2 and 3 and Table 4, Nrf2 protein expression and the Nrf2 nuclear translocation rate were increased (P<0.01) in the AD model group compared with the control group, and HO-1 protein expression was increased in the AD model group (P<0.05). Compared with the model group, the high-dose Res group showed reduced Nrf2 protein expression, increased nuclear Nrf2 protein expression, an increased nuclear translocation rate of Nrf2 (P<0.05), and an increased HO-1 protein level (P<0.05).

### 5.5. Effect of Res on the mRNA Expression of ER*α*, ER*β*, and ChAT mRNA in the AD Model

**Figure 4:**
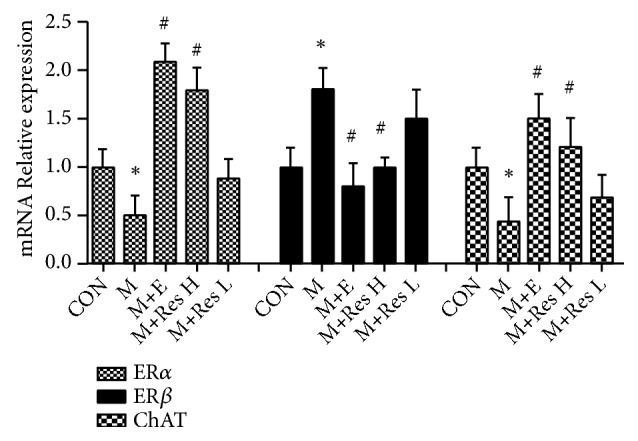
Changes in the relative mRNA expression of ER*α*, ER*β*, and ChAT in the brain tissue from each group ($\overset{-}{x}$±s, n=10). Note: compared with the CON group, *∗*P <0 .05; compared with the model group, #P <0.05.

When detecting the mRNA expression of ER*α*, ER*β*, and ChAT mRNA in the brain tissue from each group, the expression level of ER*β* in the model group was found to be significantly higher than that in the CON group, while the expression levels of ER*α* and ChAT were significantly lower. However, compared with that of the M group, the expression of ER*β* in the M+E and M+Res H groups was significantly decreased, and the mRNA expression of ER*α* and ChAT was increased in the M+E and M+Res H groups at the same time. However, there were no significant differences between the M+Res L group and M group. These results suggested that the high dose of Res could downregulate ER*β* mRNA expression and upregulate the mRNA expression of ER*α* and ChAT (Figure 4).

### 5.6. Analysis of the Protein Expression Changes of ER*α*, ER*β*, and ChAT by Western Blot Analysis

**Figure 5:**
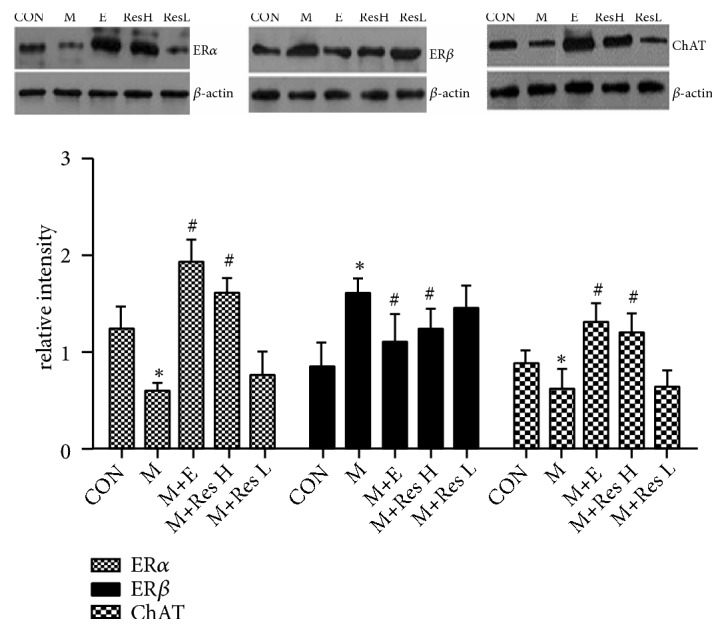
Changes in the protein expression of ER*α*, ER*β*, and ChAT. Note: compared with the CON group, *∗*P <0 .05; compared with the model group, #P <0.05.

Through experimentation, it was found that, compared with the CON group, the model group exhibited significantly increased ER*β* protein levels and significantly decreased ER*α* and ChAT protein levels. However, the expression of the ER*β* protein in the M+E and M+Res H groups was significantly lower than that in the model group, and the protein expression of ER*α* and ChAT was simultaneously increased in the M+E and M+Res H groups; however, there were no significant differences between the M+Res L group and the M group. These results suggested that the high dose of Res can downregulate ER*β* protein expression and upregulate the protein expression of ER*α* and ChAT, which is consistent with the changes in the mRNA levels (Figure 5).

### 5.7. The Effect of Res on the Expression of A*β* and Estradiol in the AD Model

**Figure 6:**
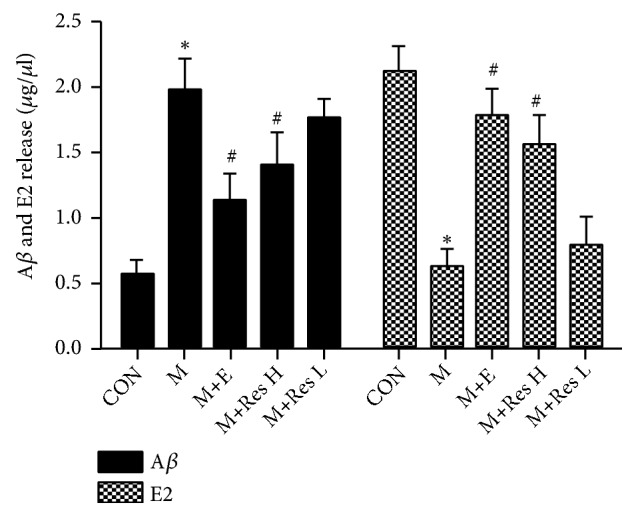
Changes in the expression of A*β* and E2 in the brain tissue from each group. Note: compared with the CON group, *∗*P <0 .01; compared with the model group, #P <0.01.

The results showed that, compared with that of the CON group, the expression of A*β* in the M group was significantly increased, and the expression of estradiol was significantly decreased in the M group. However, the expression of A*β* in the M+E and M+ResH groups decreased to varying degrees and the expression of estradiol simultaneously increased to different levels; however, there were no significant differences between the M+Res L group and the M group. These results suggested that, after the intervention with the high dose of Res, the expression of A*β* at the protein level could be downregulated while the expression of estradiol at the protein level could be upregulated (Figure 6).

## 6. Discussion

AD is a common type of dementia, and its prevalence is increasing every year [8]. Since AD is a chronic, degenerative central nervous system disease, a patient's memory will gradually decline, and cognitive function will also be impaired. However, the lack of a clearly identified pathogenic factor for AD has caused great obstacles to clinical treatment. SAMP8 mice are fast-aging mice, whose life spans are generally 12-13 months. Studies have shown that the activities of many antioxidative stress response enzymes such as SOD [9], GSH-Px [10], and catalase [11], in SAMP8 mice, decrease to different degrees, and there will also be deposition of A*β* in the brain [12]; all these effects are consistent with the pathological mechanism of AD. As a result, SAMP8 mice are widely used as an animal model for AD-related basic research. Res is a plant polyphenol that has a strong antioxidant capacity and other extensive biological effects. The results of the water maze experiment in this study show that Res can significantly improve the learning ability of mice in the model group. Oxidative stress is associated with all aspects of AD pathogenesis and is closely related to the formation of pathological features in AD [13]. Relative to normal people, AD patients exhibit increased oxidative damage, such as lipid peroxidation, and reactive carbonyl and nucleic acid oxidation, in their neurons, and the abovementioned oxidative markers are readily apparent in the fragile neurons of AD patients but not obvious in other diseases, indicating that oxidative stress responses occur earlier than other markers [4]. A study found that obvious oxidative stress can be observed in each phase of AD and that oxidative stress increased with progression [14]. Nrf2 is an important transcription factor that regulates antioxidative stress, and it is commonly expressed in various tissues and cells. Under normal conditions, the activity of the Nrf2 pathway is suppressed. However, when cells are damaged by oxidative stress, Nrf2 dissociates from its specific receptor Keap 1, enters the nucleus, and activates of antioxidation response elements (ARE), which regulates the expression of antioxidant enzyme genes [15, 16]. The antioxidant enzyme genes regulated by ARE include HO-1 and glutathione-S-transferase. Their elevated expression contributes to clearing ROS, increasing glutathione synthesis, deacidifying quinonoids, detoxifying exogenous chemicals, etc. These processes thereby protect cells from oxidative stress and maintain the dynamic balance of the intracellular partial pressure of oxygen. In this study, we showed that a high dose of Res can significantly shorten the latency and swimming distance of these model mice and improve the learning ability of mice through water maze experiment. On this basis, we further studied the effects of Res on oxidative stress and estrogen levels in the model mice. Through this study, it was found that the activities of SOD, GSH-Px, and CAT in the model mice were indeed low, but the activities of SOD, GSH-Px, and CAT were upregulated after the intervention with Res. In addition, the intervention with Res could decrease the MDA content and Nrf2 protein content in brain tissue and increase the nuclear Nrf2 content and the Nrf2 nuclear translocation rate, while continuing to upregulate HO-1 expression. An increase in HO-1 expression in brain tissue can enhance the antioxidant capacity of HO-1. The expression of HO-1 increased with the increase in the nuclear translocation of Nrf2, indicating that HO-1 is involved in the antioxidant defense mechanism in AD.

The occurrence of AD exhibits a clear gender difference. According to statistics, approximately 15% of women over the age of 65 suffer from AD, and the incidence in men is only approximately one-third of that in women. The main reason for the difference is that estrogen in the male brain can be produced by aromatase catalysis of testosterone or de novo synthesis by neurons or glial cells [17]. Clinical epidemiological studies have also found that postmenopausal women with AD are significantly higher than men of the same age, and clinical studies have indicated that estradiol replacement therapy can improve learning and memory function in AD patients [18]. Through experiments, we found that the levels of A*β* and ER*β* in the brain tissue from the model group were elevated, but the expression levels of estradiol, ER*α*, and ChAT were decreased. The intervention with Res not only reduced the deposition of A*β* and the expression of ER*β* in the brain of AD animal models but also upregulated the levels of estrogen-estradiol, its receptor ER*α*, and ChAT. Estrogen plays a neuroprotective role in the pathogenesis of AD. The decrease in estradiol levels not only makes the prevalence of AD significantly higher in older women than men but also enhances the incidence of developmental AD in women. In this study, Res increased the levels of ER*α* and ChAT, suggesting that Res has estrogen-like effects and can improve the pathogenesis of AD by regulating the expression of estrogen receptors.

This study used SAMP8 mice as research models and adopted an active ingredient in Chinese medicine, Res, as an intervention. The behavior and molecular biology studies were used for observation and detection. It was found that Res could significantly increase the antioxidant response in the AD model and increase the estrogen levels at the same time, but the mechanism needs further study. The preliminary validation of this study was that Res improved the oxidative stress response and estrogen levels in AD patients. This provides a theoretical basis for the clinical treatment of AD and provides a path for the development of Chinese medicine.

## Figures and Tables

**Table 1:** Primer sequence and amplification product size.

Gene name	Primer sequence	Amplification product (bp)
SOD	Forward 5'-CACAACTGGTTCACCGCTTG-3'	91
	Reverse 5'-GCCCAACCAGACAGAGAATGA-3'	
GSH-Px	Forward 5'-CGTGCAATCAGTTCGGACC-3'	101
	Reverse 5'-CCAGGCATCTCCCTTCCATTC-3'	
CAT	Forward 5'-CAGGAAGGCTTGCTCAGGAA-3'	81
	Reverse 5'-AGGACGGGTAATTGCCATTG-3'	
HO-1	Forward 5'-GGGTCCTCACACTCAGTTT-3'	228
	Reverse 5'-CCAGGCATCTCCCTTCCATTC-3'	
ChAT	Forward 5'-CCGGTGAACCCCTTAAGC-3'	109
	Reverse 5'-GTCGAAAAGGGACATAGC-3'	
ER*α*	Forward 5'-ACTGGCCAATCTTTCTCTGC-3'	100
	Reversed '-CAATTCATCCCCAAAGACATGGAC-3'	
ER*β*	Forward 5'-TCACTTCTGCGCTGTCTGCAGCG-3'	120
	Reversed'-CCTGGGTCGCTGTGCCAAG-3'	
*β*-actin	Forward 5'- GGGAAATCGTGCGTGACATT-3'	144
	Reverse 5'- GCGGCAGTGGCCATCTC-3'	

**Table 2:** Comparison of the learning abilities of the mice in each group.

Group	Rat number	latency time (s)	Swimming distance (cm)
Control group	10	49.88±4.19	69.7±0.35
Model group	10	64.42±6.26^*∗*^	105.5±0.25^*∗*^
M+Res H group	10	54.9±7.28^#^	71.3±0.23^#^
M+Res L group	10	62.9±6.48	99.3±0.63

Note: compared with the control group, *∗*P <0.05; compared with the model group, #P <0.05.

**Table 3:** The effect of Res on the antioxidant capacity of the AD model (±s, n=10).

Groups	SOD (U/mL)	GSH-Px (*μ*M)	CAT (U/mL)	MDA (nM)
Control group	2.92±0.28	2.88±0.19	2.97±0.35	2.19±0.17
Model group	0.62±0.17^*∗*^	0.42±0.26^*∗*^	0.55±0.25^*∗*^	3.69±0.42^*∗*^
High-group Res group	2.04±0.23^#^	1.99±0.28^#^	2.13±0.23^#^	1.97±0.57^#^
Low-dose Res group	0.96±0.33	0.79±0.19	1.03±0.15	0.91±0.22

Note: compared with the control group, *∗*P <0.05; compared with the model group, #P <0.05.

**Table 4:** Effect of Res on Nrf2 protein expression in the AD model brain tissue (±s, n=5).

Groups	Nrf2 protein (cytoplasm)	Nrf2 protein (nucleus)	Nrf2 nucleus translocation
Control group	0.17±0.03	0.25±0.05	25.74±4.94
Model group	1.25±0.23*∗∗*	0.68±0.18*∗∗*	35.76±3.92*∗∗*
Res H group	0.95±0.34#	0.85±0.12#	40.54±5.36##
Res L group	1.17±0.27	0.37±0.07	26.65±4.32

Compared with the control group, *∗∗*P<0.01; compared with the model group, #P<0.05 and ##P<0.01.

## Data Availability

Accurate data supply is hoped to be made; thus, data is welcome to be contacted by email to our correspondent author, hby616688@163.com.
